# Effect of the interpregnancy interval after early pregnancy loss on pregnancy outcomes after subsequent embryo transfer: a retrospective cohort study

**DOI:** 10.7717/peerj.20949

**Published:** 2026-03-16

**Authors:** Junrong Diao, Xinyan Wang, Ying Han, Yinfeng Zhang, Jingwen Liu, Yunshan Zhang, Haining Luo, Yuanjing Hu

**Affiliations:** 1 Clinical School of Obstetrics and Gynecology Center, Tianjin Medical University, Tianjin, China; 2 Center for Reproductive Medicine, Tianjin Central Hospital of Gynecology Obstetrics, Tianjin, China; 3 Tianjin Medical University, Tianjin, China; 4 Department of Gynecological Oncology, Tianjin Central Hospital of Gynecology Obstetrics, Tianjin, China

**Keywords:** Interpregnancy interval, Early pregnancy loss, Embryo transfer, Pregnancy

## Abstract

**Background:**

After early pregnancy loss (EPL), couples often seek counselling on how long to wait before attempting to conceive again. However, the optimal interpregnancy interval (IPI) between EPL and the next pregnancy is controversial. Additionally, studies on the impact of the IPI following a previous EPL on the population with infertility are rare. Here, we explore the relationship between the IPI after EPL and pregnancy outcomes after subsequent frozen embryo transfer (FET).

**Methods:**

In total, 859 patients were included in this retrospective study. Among these, 87 women (10.1%) had an IPI of less than 3 months, 402 (46.8%) had an IPI of 3 to 6 months, 279 (32.5%) had an IPI of 6 to 12 months, and 91 (10.6%) had an IPI of more than 12 months. The baseline characteristics of the four groups were compared and analyzed. Binary logistic regression analyses were subsequently conducted to investigate the association between the IPIs after EPL and pregnancy outcomes after subsequent FET.

**Results:**

The live birth rates of the four groups were 41.4%, 41.5%, 36.9% and 28.6%, respectively. There were no significant differences in live birth, biochemical pregnancy, clinical pregnancy, clinical pregnancy loss, or preterm birth among the four groups (*P* > 0.05). The results of the logistic regression analyses revealed that compared with an IPI of 3 to 6 months, a shorter IPI (1∼3 months) was not associated with decreased odds of live birth (adjusted OR, 1.001 [0.61–1.63]), biochemical pregnancy (adjusted OR, 0.95 [0.58–1.54]), or clinical pregnancy (adjusted OR, 0.96 [0.59–1.55]), and was not associated with an increased risk of clinical pregnancy loss (adjusted OR, 0.90 [0.41–1.97]) or preterm birth (adjusted OR, 0.87 [0.28–2.67]). However, compared with an IPI of 3 to 6 months, a longer IPI (≥ 12 months) was associated with reduced odds of a live birth (adjusted OR, 0.55 [0.32–0.93]).

**Conclusions:**

The results of this study suggest that a short IPI following the return of menstruation did not appear to be significantly associated with adverse pregnancy outcomes. However, prolonging the IPI beyond 12 months might correlate with a reduced likelihood of achieving a live birth, although further research is needed to confirm this observation.

## Introduction

Early pregnancy loss (EPL) is a common complication of assisted reproductive technology (ART) treatment and affects approximately 15% of intrauterine pregnancies (Hipp et al., 2016). This adverse pregnancy event can be physically and psychologically traumatic to infertility patients (Farren et al., 2020; Quenby et al., 2021). Most women who experience EPL want to know whether they can do anything to prevent a subsequent pregnancy loss, and often have a strong desire to seek a physician’s advice on how long they should wait before attempting to conceive again (Sharon-Weiner et al., 2021).

Most studies on the interpregnancy interval (IPI) have focused on the interval between a live birth and subsequent pregnancy (Conde-Agudelo, Rosas-Bermúdez & Kafury-Goeta, 2006; Wang et al., 2022; Shachar et al., 2016; Shachar & LD, 2012; Quinn et al., 2019). There is convincing evidence that both short (<18 months) and long IPIs (>59 months) are associated with an increased risk of adverse maternal and perinatal outcomes during the next pregnancy (Wang et al., 2022; Shachar & LD, 2012). However, the optimal IPI for the next pregnancy following EPL is controversial. Many clinicians advise patients to wait at least 3 months after EPL to reduce the likelihood of another EPL (Hurd, 2016), while the World Health Organization (WHO) recommends an IPI > 6 months after spontaneous or induced abortion (Organization, 2005). An early study in Latin America reported that a post-abortion IPI shorter than 6 months is independently associated with increased risks of adverse maternal and perinatal outcomes (maternal anaemia, premature rupture of membranes, preterm birth and low birth weight) (Conde-Agudelo et al., 2005). However, a meta-analysis (including 16 studies with a total of 1,043,840 women) revealed that an IPI less than 6 months after miscarriage did not increase the risk of adverse outcomes but did reduce the risk of further miscarriage and preterm birth during the next pregnancy (Kangatharan, Labram & Bhattacharya, 2017). Moreover, multiple studies revealed that a very short IPI (<3 months) after a miscarriage was not associated with increased risks of adverse pregnancy outcomes (Wong et al., 2015) but was more likely to be associated with a live birth (Tessema et al., 2022; DaVanzo, Hale & Rahman, 2012).

Most existing research on the IPI and subsequent pregnancy outcomes is based on naturally conceived pregnancies. Women undergoing *in vitro* fertilization (IVF) are more prone to psychological distress (Braverman et al., 2024), and compared with women who conceive naturally, those who conceive *via* IVF generally have an increased risk of adverse pregnancy outcomes (Luke, 2017). Therefore, it is necessary to conduct research on the optimal IPI after a previous EPL in the IVF population. Notably, studies that evaluate IPIs specifically among women undergoing IVF are notably insufficient. One study with a small sample size revealed that shorter intervals (median 57.5 days) were associated with a greater likelihood of having a live birth (Sharon-Weiner et al., 2021). However, a recent study published in JAMA revealed that a shorter IPI (<6 months) was associated with decreased odds of clinical pregnancy, live birth and healthy live birth and suggested delaying the next frozen embryo transfer (FET) for at least 6 months after clinical pregnancy loss (Wang et al., 2023). Therefore, further studies are needed to investigate the optimal IPI following prior EPL in patients undergoing *in vitro* fertilization (IVF).

The objective of this study was to evaluate the relationship between the length of the IPI after EPL and subsequent pregnancy outcomes in patients who underwent IVF. In this study, we attempted to address an important clinical question that could assist clinicians in providing professional advice on the interval between the preceding EPL and the start of the next embryo transfer cycle.

## Methods

### Study design and population

This was a single-center retrospective cohort study. The study included women who underwent FET after EPL occurred during a preceding embryo transfer originating from the same ovarian stimulation cycle, between January 1, 2015, and December 31, 2022. All of the data were retrieved from the electronic medical records system of the Center for Reproductive Medicine at Tianjin Central Hospital of Obstetrics and Gynecology. All couples had completed a comprehensive infertility evaluation prior to initiating the initial IVF treatment. This process included gathering the couple’s medical history, assessing the female’s serum endocrine levels, conducting an ultrasound examination, checking the patency of the fallopian tubes, performing a hysteroscopy if needed, and carrying out a routine semen analysis. All of the data were recorded in the electronic medical records system.

The interval between the date of expulsion of pregnancy tissue following EPL and the subsequent initiation of an FET cycle was defined as the interpregnancy interval (IPI) in this study. Critically, in accordance with standard clinical protocol, all FET cycles were initiated only after the resumption of at least one menstrual period. Consequently, by definition, the shortest possible IPI in our cohort is greater than one month.

The inclusion criteria were as follows: (1) The embryo transferred originated from the same ovarian stimulation cycle, and (2) for women with multiple eligible EPL–FET sequences, we included only one sequence per woman, prioritizing the first occurring one with complete follow-up data. The exclusion criteria were as follows: (1) the presence of factors affecting endometrial receptivity, including uterine malformations, intrauterine adhesions, uterine submucosal myoma, adenomyosis, or hydrosalpinx; (2) a history of recurrent pregnancy loss (defined as two or more previous spontaneous pregnancy losses); (3) a chromosomal abnormality in either member of the couple; or (4) incomplete follow-up records.

According to the length of the IPI, we divided the eligible patients into four groups: 1∼3 months group, 3 to 6 months group, 6 to 12 months group and ≥12 months group (Wang et al., 2023).

This study was approved by the Ethics Committee of Tianjin Central Hospital of Obstetrics and Gynecology (Protocol number: ZY2024001), and a waiver of informed consent was granted because this study was a retrospective analysis of deidentified data.

### Endometrial preparation and embryo transfer

Endometrial preparation protocols were customized by the treating physician based on the patient’s menstrual patterns and clinical conditions, as detailed in our previous work (Diao et al., 2024). These protocols included natural cycles, hormone replacement therapy and mild ovarian stimulation. The embryos were cryopreserved and subsequently thawed in accordance with the protocols specified by the vitrified freezing and resuscitation solution (Japan Kato), as detailed in Diao et al. (2024). The criteria for high-quality day three embryos were defined as those with 7–10 cells, uniform or slightly uneven blastomere sizes, and no fragments or fragments ≤10%, following established criteria (Desai, Rowland & Goldfarb, 2000). Blastocyst quality was assessed using the Gardner and Schoolcraft grading system (Gardner et al., 2000), with high-quality blastocysts defined as having an expansion score of ≥4, an inner cell mass grade of ≥B, and a trophectoderm grade of ≥B. Embryo transfers were performed by experienced senior physicians, with a maximum of two embryos transferred per cycle. Luteal phase support was initiated on the day of transfer and continued until 10 weeks of pregnancy if pregnancy was confirmed. Serum human chorionic gonadotropin (hCG) was measured on day 14 after FET. Clinical pregnancy was confirmed *via* transvaginal ultrasound at 6–7 weeks post-FET. Subsequent pregnancy outcomes were obtained *via* telephone follow-up by physicians or nurses and recorded in the electronic health system.

### Outcome measures

The primary outcome measure was the live birth rate. The secondary outcomes were biochemical pregnancy, clinical pregnancy, clinical pregnancy loss, and preterm birth. Biochemical pregnancy was defined as a positive pregnancy test result indicating a serum hCG concentration of 25 IU/L or higher, either 12 days after FET for blastocyst embryos or 14 days after FET for cleavage-stage embryos. Clinical pregnancy was defined as the presence of a gestational sac visible on transvaginal ultrasound. Clinical pregnancy loss was defined as the loss of a clinically recognized intrauterine pregnancy before 24 weeks of gestation (Wang et al., 2023). Preterm birth was defined as delivery at <37 weeks of gestation. Live birth was defined as the delivery of any neonate exhibiting signs of life at or beyond 24 weeks of gestation.

### Observation indices and calculation method

The basic characteristics of the patients in each group (such as age, body mass index (BMI), causes of infertility, previous miscarriage and childbirth counts), the IVF process (number of oocytes retrieved, fertilization method, *etc.*), the FET process (number of embryos transferred, number of high-quality embryos transferred, endometrial thickness on the day of embryo transfer, *etc.*), and the outcomes of embryo transfer (such as the biochemical pregnancy rate, clinical pregnancy rate, pregnancy loss rate, preterm birth rate, and live birth rate, *etc.*) were compared.

Biochemical pregnancy rate = (number of biochemical pregnancy cycles/number of transfer cycles) × 100%. Clinical pregnancy rate = (number of clinical pregnancy cycles/number of transfer cycles) × 100%. Clinical pregnancy loss rate = (number of pregnancy loss cycles/number of clinical pregnancy cycles) × 100%. Live birth rate = (number of live birth cycles/number of transfer cycles) × 100. Preterm birth rate = (number of preterm birth cycles/number of live birth cycles) × 100%.

### Statistical analyses

Continuous variables are expressed as the means ± SDs and were assessed for normality. The Kolmogorov–Smirnov test was used to evaluate the normality of the data. A normal distribution diagram (frequency histogram) was used to visually check whether the data were approximately normally distributed. Normally or approximately normally distributed data were compared using ANOVA, whereas skewed data were compared *via* the Kruskal–Wallis test. Categorical variables were summarized as counts and percentages, and Pearson’s chi-square test or Fisher’s exact test, when appropriate, was used to compare differences between groups. The Cochran–Armitage trend test was used to evaluate the linear relationship between ordered IPI categories (1∼3 months, 3∼6 months, 6∼12 months, and ≥12 months) and live birth rates. Binary logistic regression analysis was conducted to assess the impact of various IPIs on FET outcomes. Crude and adjusted odds ratios (ORs) along with 95% confidence intervals (CIs) are presented. Potential confounders, such as female age at the time of oocyte pick up (OPU), body mass index (BMI), number of previous pregnancies and deliveries, number of previous embryo transfer cycles, diagnosis of polycystic ovary syndrome (PCOS), gestational age at the preceding EPL, means used to terminate the preceding EPL, endometrial preparation protocols for FET, endometrial thickness, developmental stage of the transferred embryo, number of embryos transferred, and the transfer of ≥1 good-quality embryo, were incorporated into the logistic regression model. Continuous covariates were modelled as linear terms in their original scale, assuming a linear relationship with the log-odds of the outcome. Confounders included in the logistic regression model were selected based on established clinical relevance and their association with outcomes in the literature (*e.g.*, maternal age, BMI, embryo quality) and statistically significant differences (*P* < 0.05) in the distribution of variables across the four groups. To assess the robustness of our findings against potential collinearity and overadjustment, a minimally adjusted model (adjusted only for maternal age and embryo quality, the most essential confounders) was performed as a sensitivity analysis, in parallel with the fully adjusted model. As multiple statistical tests were performed across different pregnancy outcomes, there is a potential for inflation of type I error. Box–Tidwell tests confirmed linearity in the logit scale (all interaction terms between continuous covariates and their log-transformed values had *P* > 0.05). Multicollinearity was assessed using the variance inflation factor (VIF), and all the variables demonstrated a VIF of < 5, indicating the absence of substantial multicollinearity. The overall model fit was evaluated with the Hosmer–Lemeshow goodness-of-fit test, which revealed no evidence of poor fit (*p* > 0.05). Statistical analysis was performed with SPSS (version 26.0. SPSS, Inc., Chicago, IL, USA). All tests were two-sided, and *P* < 0.05 was considered to indicate statistical significance.

## Results

**Figure 1:**
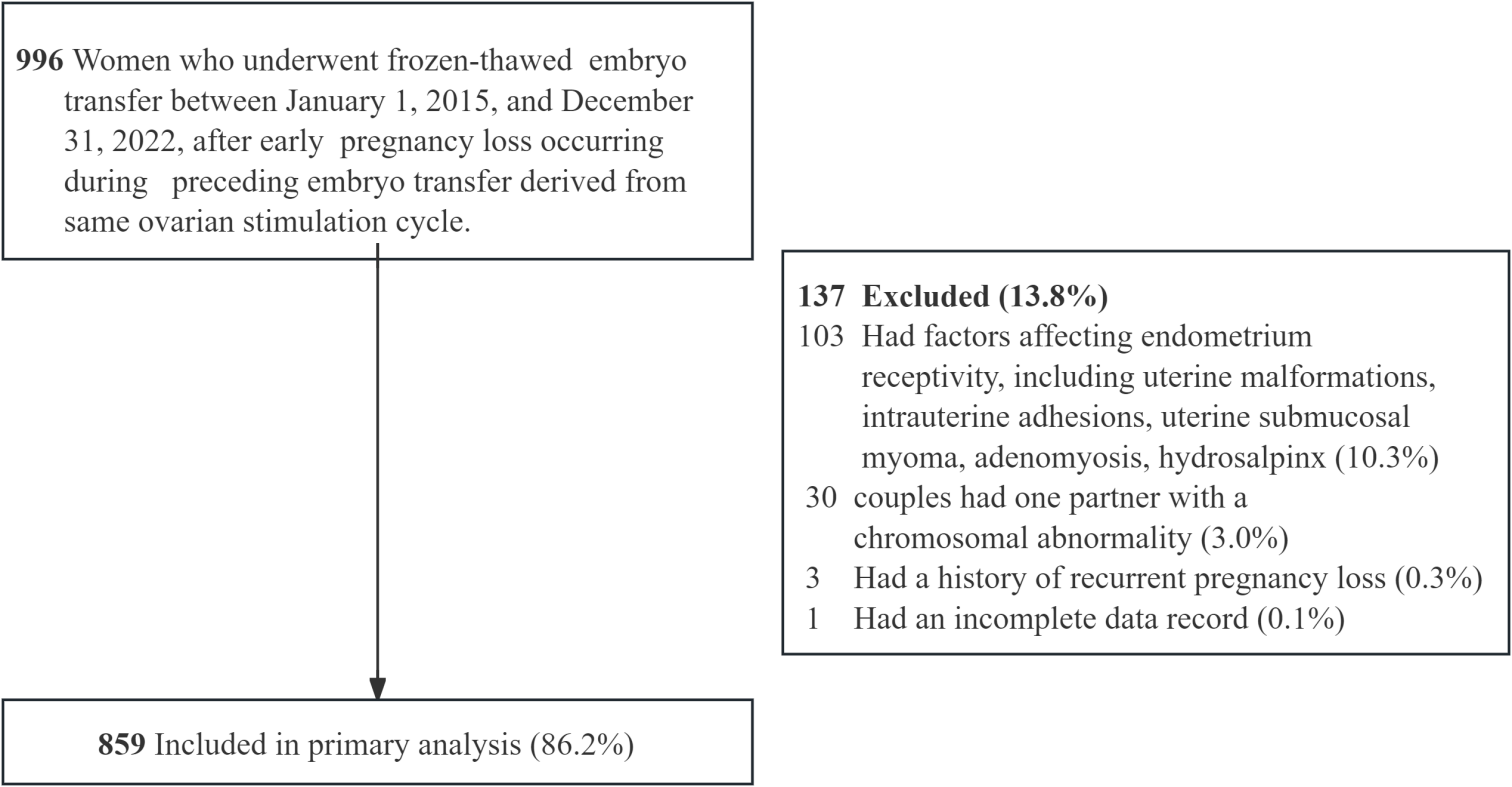
Flowchart of enrolled patients.

A total of 859 patients were included in this study (Fig. 1). The distribution of IPIs among the participants was as follows: 87 women (10.1%) had an IPI of 1 to 3 months, 402 (46.7%) had an IPI of 3 to 6 months, 279 (32.5%) had an IPI of 6 to 12 months, and 91 (10.6%) had an IPI of more than 12 months. The baseline characteristics of the female participants in the four groups are presented in Table 1. The general characteristics, including female age at FET, BMI, and main causes of infertility, did not significantly differ among the four groups. Similarly, there were no significant differences in ovarian reserve-related factors, such as female age at OPU, baseline FSH level or number of retrieved oocytes. Women with shorter IPIs (1∼3 months or 3∼6 months) were more likely to undergo one embryo transfer cycle than those with longer IPIs (6∼12 months or ≥12 months) were (75.9% or 70.1% *vs.* 63.1% or 59.3%, respectively). Women with a short IPI (1∼3 months) were more likely to have experienced the preceding EPL at an earlier gestational age (59.02 ± 12.65 days *vs.* 64.01 ± 12.37 days, 65.71 ± 12.51 days or 66.07 ± 13.10 days) and were less likely to have undergone surgical evacuation for the preceding EPL (10.3% *vs.* 26.1%, 43.0% or 44.0%) than those with longer IPIs (3∼6 months, 6∼12 months or ≥12 months). The distribution of previous pregnancies differed significantly among the four groups (*P*  <  0.05).

**Table 1:** Baseline demographics of patients in the study.

**Characteristic**	1∼3 months (*n* = 87)	3∼6 months (*n* = 402)	6∼12 months (*n* = 279)	≥12 months (*n* = 91)	*P* value
Female age at OPU (years)	32.49 ± 4.70	31.96 ± 4.29	32.37 ± 4.09	31.48 ± 3.68	0.235
Female age at FET (years)	33.07 ± 4.60	32.75 ± 4.25	33.59 ± 4.08	33.49 ± 3.71	0.061
Body mass index (kg/m^2^ )	22.50 ± 3.21	22.81 ± 3.13	22.87 ± 3.06	23.10 ± 3.36	0.644
Main causes of infertility, *n* (%)					0.251
Tubal factor	44 (50.6%)	219 (54.5%)	163 (58.4%)	44 (48.4%)	
Ovarian factor	8 (9.2%)	53 (13.2%)	40 (14.3%)	17 (18.7%)	
Male factor	27 (31.0%)	110 (27.4%)	61 (21.9%)	27 (29.7%)	
Unexplained infertility	8 (9.2%)	20 (5.0%)	15 (5.4%)	3 (3.3%)	
Diagnosis of PCOS	10 (11.5%)	43 (10.7%)	35 (12.5%)	18 (19.8%)	0.126
Diminished ovarian reserve	5 (5.7%)	29 (7.2%)	18 (6.5%)	7 (7.7%)	0.936
FSH level (mIU/ml)	6.31 ± 2.10	6.44 ± 1.93	6.41 ± 2.10	6.43 ± 1.88	0.951
No. of retrieved oocytes, *n*	17.46 ± 10.63	17.15 ± 8.03	17.84 ± 8.36	16.31 ± 7.73	0.464^*^
Fertilization method, *n* (%)					0.887
IVF	51 (58.6%)	249 (61.9%)	170 (60.9%)	53 (58.2%)	
ICSI	36 (41.4%)	153 (38.1%)	109 (39.1%)	38 (41.8%)	
No. of previous clinical pregnancy, *n* (%)					0.001
1	50 (57.5%)	229 (57%)	137 (49.1%)	52 (57.1%)	
2	17 (19.5%)	81 (20.1%)	96 (34.4%)	25 (27.5%)	
≥3	20 (23%)	92 (22.9%)	46 (16.5%)	14 (15.4%)	
No. of previous delivery, *n* (%)					0.051
0	69 (79.3%)	338 (84.1%)	231 (82.8%)	85 (93.4%)	
≥1	18 (20.7%)	64 (15.9%)	48 (17.2%)	6 (6.6%)	
History of cesarean delivery, *n* (%)	8 (9.2%)	33 (8.2%)	28 (10%)	2 (2.2%)	0.129
No. of previous embryo transfer cycles, *n* (%)					0.034
1	66 (75.9%)	282 (70.1%)	176 (63.1%)	54 (59.3%)	
2	19 (21.8%)	93 (23.1%)	72 (25.8%)	26 (28.6%)	
≥3	2 (2.3%)	27 (6.7%)	31 (11.1%)	11 (12.1%)	
Gestational age of preceding EPL, *d*	59.02 ± 12.65	64.01 ± 12.37	65.71 ± 12.51	66.07 ± 13.10	<0.001
Surgical evacuation for the preceding EPL, *n* (%)	9 (10.3%)	105 (26.1%)	120 (43.0%)	40 (44.0%)	<0.001

**Notes.**

OPU oocyte pick up FET frozen embryo transfer PCOS polycystic ovary syndrome EPL early pregnancy loss

Continuous variables were presented as mean and (SD). ANOVA or Kruskal-Wallis test was performed as appropriate.

* Kruskal–Wallis test.

Categorical variables were presented as n(%). Pearson’s Chi-square test was used to compare differences between groups. *P* < 0.05 was considered statistically significant.

The clinical features of the frozen-thawed embryo transfer cycles in the four groups are presented in Table 2. There was a correlation between the IPIs and several key factors. Specifically, increases in the IPI were correlated with a decrease in the proportion of patients who underwent endometrial preparation with natural cycle regimens (80.5%, 81.8%, 73.5%, and 65.9%), a reduction in endometrial thickness (9.54 ± 1.34, 9.43 ± 1.62, 9.15 ± 1.57, and 8.86 ± 1.40, *P* < 0.05), and an increase in the proportion of patients with an endometrial thickness ≤7 mm (0, 1.5%, 5.0%, and 5.5%, respectively). A thin endometrium (≤7 mm) was observed in only 2.9% of the reported cases (25/859). Gestational age and surgical evacuation of EPL may impact endometrial thickness, while irregular menstrual cycles in women with polycystic ovary syndrome (PCOS) or diminished ovarian reserve (DOR) can also influence the endometrial preparation regimen. Women with a long IPI (≥12 months) were more likely to undergo FET of blastocyst embryos.

**Table 2:** Clinical characteristics of this frozen-thawed embryo transfer cycle.

**Characteristic**	1∼3 months (*n* = 87)	3∼6 months (*n* = 402)	6∼12 months (*n* = 279)	≥12 months (*n* = 91)	*P* value
Endometrial preparation, *n* (%)					<0.001
Natural cycle	70 (80.5%)	329 (81.8%)	205 (73.5%)	60 (65.9%)	
Hormonal replacement therapy	11 (12.6%)	47 (11.7%)	64 (22.9%)	18 (19.8%)	
Stimulated regimen	6 (6.9%)	26 (6.5%)	10 (3.6%)	13 (14.3%)	
Endometrial thickness before FET (mm)	9.54 ± 1.34	9.43 ± 1.62	9.15 ± 1.57	8.86 ± 1.40	0.001^*^
Endometrial thickness ≤7 mm	0	6 (1.5%)	14 (5.0%)	5 (5.5%)	
No. of embryos transferred, *n* (%)					0.178
1	13 (14.9%)	57 (14.2%)	53 (19.0%)	20 (22.0%)	
2	74 (85.1%)	345 (85.8%)	226 (81.0%)	71 (78.0%)	
Embryo developmental stage, *n* (%)					<0.001
Cleavage	80 (92%)	356 (88.6%)	235 (84.2%)	66 (72.5%)	
Blastocyst	7 (8%)	46 (11.4%)	44 (15.8%)	25 (27.5%)	
Transfer ≥1 good-quality embryo, *n* (%)	59 (67.8%)	276 (68.7%)	180 (64.5%)	58 (63.7%)	0.637

**Notes.**

Continuous variables were presented as mean and (SD). ANOVA or Kruskal–Wallis test was performed as appropriate.

* Kruskal–Wallis test.

Categorical variables were presented as n(% ). Pearson’s Chi-square test was used to compare differences between groups. *P* < 0.05 was considered statistically significant.

**Figure 2:**
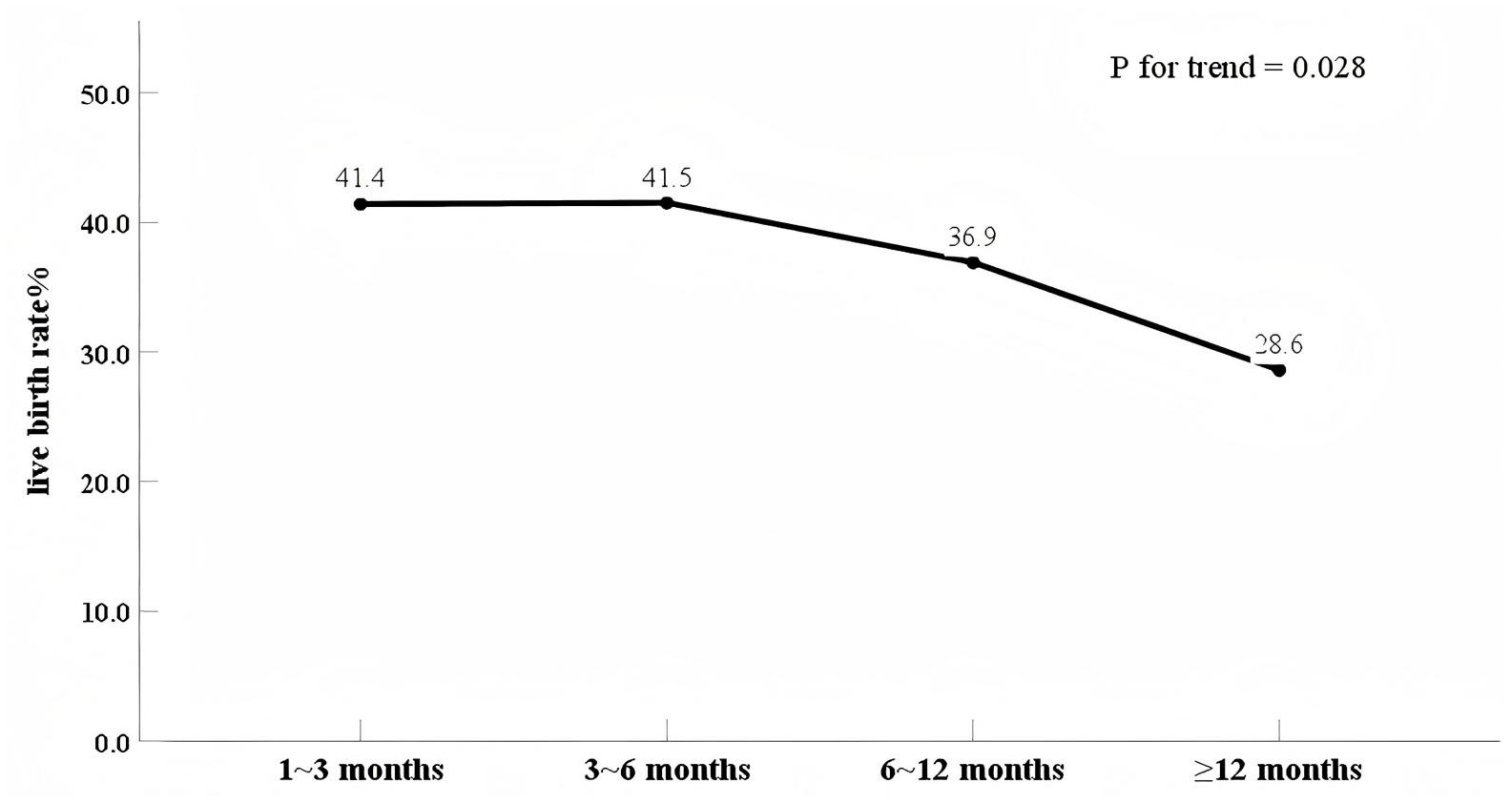
The linear relationship between the ordered IPI categories and live birth rates.

Comparisons of pregnancy outcomes among the 4 groups are shown in Table 3. The live birth rates were 41.4% (1∼3 months), 41.5% (3–6 months), 36.9% (6–12 months), and 28.6% (≥12 months) across the four IPI groups, with an overall *P* value of 0.114. There were no significant differences in biochemical pregnancy, clinical pregnancy, clinical pregnancy loss, or preterm birth rates among the four groups. To further characterize the directional pattern, we performed a Cochran–Armitage trend test to assess the linear relationship between the ordered IPI categories and live birth rates. This test revealed a statistically significant trend towards decreasing live birth rates with increasing IPIs (*P* = 0.028). To visualize this trend, we generated a line graph (Fig. 2) depicting the live birth rates across IPI groups. As shown in Table 3, the clinical pregnancy loss rates were 20.0% (1∼3 months), 19.7% (3–6 months), 24.3% (6–12 months), and 29.7% (≥12 months) across the four IPI groups, with an overall *P* value of 0.488. Statistical trend analysis revealed a nonsignificant trend towards increasing clinical pregnancy loss rates with longer IPIs (*P* = 0.156).

**Table 3:** Pregnancy outcomes according to categorical IPIs.

**Outcomes**	1∼3 months (*n* = 87)	3∼6 months (*n* = 402)	6∼12 months (*n* = 279)	≥12 months (*n* = 91)	*P* value
SET, *n/N* (%)	13/87 (14.9%)	57/402 (14.2%)	53/279 (19.0%)	20/91 (22.0%)	0.178
DET, *n/N* (%)	74/87 (85.1%)	345/402 (85.8%)	226/279 (81.0%)	71/91 (78.0%)	0.178
Live birth, *n/N* (%)	36/87 (41.4%)	167/402 (41.5%)	103/279 (36.9%)	26/91 (28.6%)	0.114
Singleton, *n* (%)	25 (69.4%)	132 (79%)	81 (78.6%)	23 (88.5%)	0.345
Twin, *n* (%)	11 (30.6%)	35 (21%)	22 (21.4%)	3 (11.5%)	
Biochemical pregnancy, *n/N* (%)	49/87 (56.3%)	232/402 (57.7%)	164/279 (58.8%)	41/91 (45.1%)	0.128
Clinical pregnancy, *n/N* (%)	45/87 (51.7%)	208/402 (51.7%)	136/279 (48.7%)	37/91 (40.7%)	0.274
Clinical pregnancy loss, *n/N* (%)	9/45 (20.0%)	41/208 (19.7%)	33/136 (24.3%)	11/37 (29.7%)	0.488
Preterm birth, *n/N* (%)	4/36 (11.1%)	22/167 (13.2%)	16/103 (15.5%)	2/26 (7.7%)	0.705^*^

**Notes.**

SET Single embryo transfer DET Double embryo transfer

Categorical variables were presented as n(%). Pearson’s chi-square test or Fisher’s exact test was used to compare differences between groups. *P* < 0.05 was considered statistically significant.

* Fisher’s exact test.

The results of logistic regression analyses, shown in Table 4, reveal that compared with an IPI of 3 to 6 months, a shorter IPI (1∼3 months) was not associated with decreased odds of a live birth (adjusted OR, 1.001 [0.61–1.63]), biochemical pregnancy (adjusted OR, 0.95 [0.58–1.54]), or clinical pregnancy (adjusted OR, 0.96 [0.59–1.55]), and was not associated with an increased risk of clinical pregnancy loss (adjusted OR, 0.90 [0.41–1.97]) or preterm birth (adjusted OR, 0.87 [0.28–2.67]). Compared with an IPI of 3∼6 months, a longer IPI (6∼12 months) was not associated with a live birth (adjusted OR, 0.86 [0.62–1.20]), biochemical pregnancy (adjusted OR, 1.12 [0.81–1.55]), clinical pregnancy (adjusted OR, 0.98 [0.71–1.36]), clinical pregnancy loss (adjusted OR, 1.36 [0.82–2.25]), or preterm birth (adjusted OR, 1.02 [0.51–2.03]). However, compared with an IPI of 3∼6 months, a longer IPI (≥12 months) was associated with reduced odds of a live birth (adjusted OR, 0.55 [0.32–0.93]). Potential confounders, such as female age at the time of OPU, BMI, number of previous pregnancies and deliveries, number of previous embryo transfer cycles, diagnosis of PCOS, gestational age at the preceding EPL, methods used to terminate the preceding EPL, endometrial preparation protocols for FET, endometrial thickness, developmental stage of the transferred embryo, number of embryos transferred, and the transfer of ≥1 good-quality embryo, were incorporated into the logistic regression model. For complete transparency, the full results of this adjusted model, including adjusted odds ratios for all covariates, are presented in the Table S1. A sensitivity analysis using a minimally adjusted model (adjusted only for female age at the time of OPU and embryo quality, the most essential confounders) confirmed the robustness of the primary finding. The association between an IPI of ≥12 months and reduced odds of live birth remained significant (adjusted OR, 0.56 [0.34–0.93]). The fully adjusted model and sensitivity models are presented in Table S2.

**Table 4:** Crude and adjusted odds ratios of pregnancy outcomes according to categorical IPIs.

**Outcomes**	1∼3 months (*n* = 87)	3∼6 months (*n* = 402)	6∼12 months (*n* = 279)	≥12 months (*n* = 91)
	OR (95% CI)	OR (95% CI)	OR (95% CI)	OR (95% CI)
Live birth				
Crude	0.99 (0.62–1.59)	1 [Reference]	0.82 (0.60–1.12)	0.56 (0.34–0.92)^*^
Adjusted	1.001 (0.61–1.63)	1 [Reference]	0.86 (0.62–1.20)	0.55 (0.32–0.93)^*^
Biochemical pregnancy				
Crude	0.94 (0.59–1.50)	1 [Reference]	1.04 (0.76–1.42)	0.60 (0.38–0.95)^*^
Adjusted	0.95 (0.58–1.54)	1 [Reference]	1.12 (0.81–1.55)	0.60 (0.36-0.97)^*^
Clinical pregnancy				
Crude	0.99 (0.62–1.58)	1 [Reference]	0.88 (0.65–1.20)	0.63 (0.40–1.01)
Adjusted	0.96 (0.59–1.55)	1 [Reference]	0.98 (0.71–1.36)	0.68 (0.42–1.12)
Clinical pregnancy loss				
Crude	1.01 (0.47–2.17)	1 [Reference]	1.18 (0.72–1.92)	1.21 (0.59–2.45)
Adjusted	0.90 (0.41–1.97)	1 [Reference]	1.36 (0.82–2.25)	1.52 (0.72–3.20)
Preterm birth				
Crude	0.83 (0.27–2.48)	1 [Reference]	1.05 (0.54–2.03)	0.38 (0.09–1.68)
Adjusted	0.87 (0.28–2.67)	1 [Reference]	1.02 (0.51–2.03)	0.32 (0.07–1.44)

**Notes.**

* *P* < 0.05 (adjusted for female age at the time of OPU, BMI, No. of previous pregnancies and deliveries, No.of previous embryo transfer cycles, diagnosis of PCOS, gestational age at the preceding EPL, means used to terminate the preceding EPL, endometrial preparation protocols for FET, endometrial thickness, developmental stage of the transferred embryo, No.of embryos transferred, and the transfer of ≥1 good-quality embryo).

Given the clinical interest in the shortest interpregnancy intervals (IPIs), we conducted a detailed examination of outcomes in these subgroups. Among the eight patients with an IPI of 1 to 2 months, 4 (50.0%) achieved a live birth, and no clinical pregnancy losses were recorded (see Table S3). To formally assess the association in a larger short-interval group, we performed a sensitivity analysis comparing the 2∼3 months IPI group to the 3∼6 months reference group. This analysis confirmed that an IPI of 2∼3 months was not associated with a significant difference in the odds of live birth (adjusted OR, 0.94 [0.56−1.57]) or clinical pregnancy loss (adjusted OR, 1.003 [0.46−2.20]) compared to the reference group (Table S4).

We conducted stratified analyses to examine whether the association between IPIs and live birth was consistent across key patient and treatment characteristics. The results are detailed in Table 5. There was no significant difference in the short IPI (1∼3 months) group compared with the IPI of 3∼6 month IPI group in any subgroup. Among women aged <35 years, those with an IPI of ≥12 months had a significantly lower odds of live birth than the reference group (3∼6 months IPI) did (adjusted OR, 0.51 [0.28–0.92]). In contrast, this association was not statistically significant among women aged ≥35 years (adjusted OR, 0.67 [0.20–2.22]). The reduced odds of live birth associated with a long IPI (≥12 months) were also pronounced in women who underwent surgical evacuation for EPL (adjusted OR, 0.38 [0.16–0.93]) and in those who had two embryos transferred (adjusted OR, 0.54 [0.30–0.97]). In summary, stratified analysis revealed that short pregnancy intervals (1∼3 months) did not significantly differ from the reference group in any subgroup, whereas the adverse association between long pregnancy intervals and live birth was most evident in young women and specific clinical scenarios.

**Table 5:** Stratified analyses of the association between categorical IPIs and live births.

**Stratified Factors**	1∼3 months	3∼6 months	6∼12 months	≥12 months
	aOR (95% CI)	aOR (95% CI)	aOR (95% CI)	aOR (95% CI)
Female age at the time of OPU				
<35 years	1.41 (0.78–2.55)	1 [Reference]	0.83 (0.56–1.23)	0.51 (0.28–0.92)^*^
≥35 years	0.41 (0.15–1.15)	1 [Reference]	0.92 (0.48–1.74)	0.67 (0.20–2.22)
Means of termination for the preceding EPL				
Non-surgical evacuation	1.08 (0.64–1.82)	1 [Reference]	0.93 (0.62–1.41)	0.68 (0.35–1.32)
Surgical evacuation	0.75 (0.17–3.31)	1 [Reference]	0.72 (0.41–1.27)	0.38 (0.16-0.93)^*^
No. of previous embryo transfer cycles				
1	0.79 (0.44–1.41)	1 [Reference]	0.91 (0.61–1.36)	0.60 (0.31–1.15)
≥2	2.02 (0.74–5.55)	1 [Reference]	0.82 (0.45–1.49)	0.54 (0.21–1.35)
No. of embryos transferred				
1	0.58 (0.12–2.72)	1 [Reference]	1.01 (0.38–2.66)	0.44 (0.09–1.98)
2	1.06 (0.63–1.80)	1 [Reference]	0.87 (0.61–1.24)	0.54 (0.30-0.97)^*^
Embryo developmental stage				
Cleavage	1.01 (0.60–1.68)	1 [Reference]	0.84 (0.59–1.19)	0.61 (0.34–1.09)
Blastocyst	1.58 (0.23–10.69)	1 [Reference]	1.16 (0.41–3.26)	0.69 (0.16–2.83)
Transfer of ≥1 good-quality embryo				
Yes	1.05 (0.59–1.90)	1 [Reference]	0.78 (0.52–1.16)	0.55 (0.29–1.02)
No	0.94 (0.36–2.46)	1 [Reference]	1.12 (0.60–2.06)	0.59 (0.21–1.63)

**Notes.**

* *P* < 0.05 (adjusted for female age at the time of OPU, BMI, No. of previous pregnancies and deliveries, No.of previous embryo transfer cycles,diagnosis of PCOS, gestational age at the preceding EPL, means used to terminate the preceding EPL, endometrial preparation protocols for FET, endometrial thickness, developmental stage of the transferred embryo, No.of embryos transferred, and the transfer of ≥1 good-quality embryo).

In this study, 31.9% (274/859) of the patients who underwent surgery for previous pregnancy loss. Logistic regression analysis was performed to further evaluate the impact of surgical evacuation on live birth rates following subsequent FET. After adjusting for potential confounding factors, logistic regression analysis did not reveal any adverse effects of surgical evacuation of previous EPL on the live birth rate after subsequent FET (adjusted OR, 1.01 [0.74–1.39]).

## Discussion

Compared with an IPI of 3 to 6 months between a preceding EPL and a subsequent FET, the results of this study revealed that a short IPI of 1 to 3 months was not associated with a decreased likelihood of achieving a clinical pregnancy or live birth, nor did it increase the risk of clinical pregnancy loss or preterm birth after FET. Conversely, an IPI exceeding 12 months was linked to a reduced likelihood of a live birth, although a limited number of women were included.

Advice given to women regarding the optimal IPI following EPL remains controversial. One study conducted in Latin America revealed that an IPI of less than 6 months is independently associated with increased risks of maternal anemia, premature rupture of membranes, low birth weight, and preterm delivery (Conde-Agudelo et al., 2005). Additionally, a heightened risk of preterm delivery in pregnancies following a short IPI (<6 months) after induced abortions was also observed in Finland (Männistö et al., 2017). A recent study published in JAMA focused on IVF populations and revealed that a shorter IPI (<6 months) was associated with decreased odds of achieving a clinical pregnancy or live birth but was not associated with an increased risk of preterm birth, low birth weight or small for gestational age after the next embryo transfer (Wang et al., 2023). That study suggested delaying the next FET for at least 6 months following clinical pregnancy loss. One potential explanation is that the physical and psychological trauma from a previous pregnancy loss may necessitate an adequate period for full physiological recovery (Wang et al., 2023). These traumas include endometrial damage, alterations in the uterine microbiome caused by termination of a previous pregnancy (Ozgur et al., 2018), and high levels of posttraumatic stress, anxiety and depression after EPL (Farren et al., 2020; Bapayeva et al., 2021).

However, some studies fail to substantiate these findings (Kangatharan, Labram & Bhattacharya, 2017; Love et al., 2010; Sholapurkar, 2010; El Behery et al., 2013). A prospective study of 4,619 Egyptian women with a history of spontaneous abortion during their first pregnancy revealed that compared with those who conceived more than 12 months after their first spontaneous abortion, women who conceived within 6 months had a higher likelihood of achieving a live birth and a lower risk of preterm delivery and low birth weight (El Behery et al., 2013). The results of a meta-analysis of sixteen studies suggested that an IPI of less than 6 months following miscarriage was not associated with increased risks of stillbirth, preeclampsia, or low birth weight. Instead, it was correlated with reduced risks of miscarriage and preterm delivery (Kangatharan, Labram & Bhattacharya, 2017). Some studies have suggested that a short IPI of less than 3 months is not associated with adverse pregnancy outcomes (Wong et al., 2015; Tessema et al., 2022; DaVanzo, Hale & Rahman, 2012; Schliep et al., 2016; Sundermann et al., 2017; Shachar et al., 2016). Wong et al. (2015) reported that an IPI ≤3 months after a prior pregnancy loss is not associated with a lower rate of live birth and appears to be comparable to an IPI > 3 months (aRR: 1.07, 95% CI [0.98–1.16]). A large cohort study from Norway suggested that conceiving within 3 months after clinical pregnancy loss is not associated with increased risks of adverse pregnancy outcomes, such as preterm birth, small for gestational age, large for gestational age, preeclampsia, or gestational diabetes mellitus (Tessema et al., 2022). Therefore, they suggested that delaying conception for 3–6 months following EPL may not be warranted.

In accordance with the findings of these natural conception studies, we did not observe any associations between a short IPI of 1∼3 months following a previous EPL and a decreased risk of live birth or an increased risk of clinical pregnancy loss and preterm delivery. The live birth rate in patients with an IPI of 1∼3 months was comparable to that in patients with an IPI of 3 to 6 months (41.4% *vs.* 41.5%), as were the rates of biochemical pregnancy, clinical pregnancy, clinical pregnancy loss and preterm birth. These results remained consistent even in the logistic regression analysis and after adjustments were made for confounding factors (adjusted OR, 1.001 [0.61–1.63] for live birth). We found no evidence of harm in the 1∼2 months IPI group, which demonstrated a 50% live birth rate without any miscarriages. Furthermore, a formal sensitivity analysis of the 2∼3 months IPI group reinforced this, showing no statistically significant increase in the risk of pregnancy loss or reduction in live birth rates. Subgroup analysis, stratified by female age at OPU, method of pregnancy termination for the preceding EPL, number of previous embryo transfer cycles, number of embryos transferred, embryo developmental stage and transfer of ≥1 good-quality embryo to assess the live birth rate revealed no significant difference in the short IPI (1∼3 months) group compared with the IPIs of 3–6 month IPI group in any subgroup. This suggests that once endometrial shedding and regeneration have been confirmed, even short IPI appear to be a safe and viable option.

In addition, we observed a significant trend towards decreasing live birth rates with increasing IPIs, with values of 41.4% (1∼3 months), 41.5% (3–6 months), 36.9% (6–12 months), and 28.6% (≥12 months) across groups. Despite this consistent trend, pairwise comparisons across IPI groups failed to reach statistical significance (overall *P* = 0.114). The limited number of women included may have influenced these results, as the studies were under powered to detect a significant difference between the groups. After controlling for potential confounding variables, the difference in live birth rates between a longer IPI (≥12 months) and an IPI of 3 to 6 months was found to be significant (adjusted OR, 0.55 [0.32–0.93]). Previous research has also indicated that prolonging the IPI beyond 12 months does not confer any benefits to pregnancy outcomes (Wong et al., 2015; DaVanzo, Hale & Rahman, 2012; Love et al., 2010; El Behery et al., 2013). El Behery et al. (2013) reported that women with an IPI >12 months had the highest risks of ectopic pregnancy and termination and were less likely to have a live birth during their second pregnancy. Wong et al. (2015) reported that women with a longer IPI had decreased rates of live births and increased rates of peri-implantation losses.

Several factors could explain our findings. First, it is plausible that women experiencing EPL would not reach a point of nutritional depletion, as EPL typically occurs within the first 12 weeks (Smits & Essed, 2001). Furthermore, according to one hypothesis, EPL could contribute to maternal growth-supporting capacities, such as increased uterine blood flow and other physiological and anatomical adaptations of the reproductive system (Zhu, Nangle & Horan, 1999), thereby potentially enhancing the success of subsequent pregnancies (Sundermann et al., 2017). Additionally, it is hypothesized that pregnancy may enhance the functional capacity of the reproductive system; however, this “protection” diminishes over time, resulting in pregnancies following long IPIs that are similar to first pregnancies (Zhu, Nangle & Horan, 1999). This may explain the adverse effects of long IPIs on pregnancy outcomes. Finally, individuals undergoing IVF often have a strong desire to achieve pregnancy and seek to expedite an embryo transfer following a pregnancy loss. We hypothesize that transplantation may be delayed in some patients because of post-EPL pathological conditions, such as uterine adhesions, infections, and inflammation, which require time for treatment and recovery. These conditions may lead to irreversible damage, reducing fertility again and impacting pregnancy outcomes following subsequent embryo transfer.

Our findings also indicate that surgical evacuation when used as a treatment for EPL does not impact the live birth rate of subsequent FETs, which is consistent with previous findings. Tamir et al. (2015) and Meng et al. (2019) reported that there was no significant difference in the pregnancy outcomes of subsequent embryo transfer between women who underwent surgical evacuation following miscarriage and those who did not. However, in our study, women with a long IPI were more likely to undergo surgical evacuation of the preceding EPL and were more likely to have a thin endometrium before FET. Surgical management may be associated with complications such as bleeding, infection, endometrial trauma, and intrauterine adhesions, all of which can lead to a reduction in endometrial thickness (Meng et al., 2019; Nanda et al., 2012; Kasius et al., 2014). A meta-analysis indicated that endometrial thickness has limited predictive value for identifying women with a low likelihood of conceiving after IVF. The commonly cited cut-off of seven mm is associated with reduced pregnancy rates but is not frequently observed (the incidence is only 2.4%) (Kasius et al., 2014). Our study revealed a similar prevalence of thin endometrium (2.9%).

Research on the role of the IPI following a previous EPL in the population with infertility is limited. Our study provides valuable clinical insights for evaluating the association between the IPI and subsequent pregnancy outcomes in the IVF population. Furthermore, we collected data on women’s ages at egg retrieval, adjusting for the potential effects of age on embryo quality. However, this study was limited by its single-center and retrospective design. The small sample size, particularly for patients with IPIs of 1∼3 months and ≥12 months, may have reduced the ability to detect significant differences in pregnancy outcomes. Furthermore, the high number of previous embryo transfer cycles, the large proportion of single-embryo transfers, and the increased proportion of blastocyst transfers in the female population with IPIs ≥12 months may also influence FET prognosis, despite stratified analysis of these factors and adjustments for multiple confounding factors. Although we adjusted for embryo quality using robust clinical proxies (embryo developmental stage and the transfer of ≥1 good-quality embryo), a more detailed morphological analysis was precluded by sample size constraints. Furthermore, embryo grading itself is subject to inherent biological heterogeneity and interobserver variation. While we employed standard laboratory protocols to minimize this variation, we acknowledge that residual confounding due to unmeasured aspects of embryo quality remains possible. Additionally, maternal comorbidities such as preeclampsia and gestational diabetes, as well as other perinatal outcomes including low birth weight and small or large for gestational age, were not measured in this study because of the limited sample size. Finally, we included the number of previous clinical pregnancies as an indicator, without distinguishing between miscarriage, ectopic pregnancy, and midterm induced labor. The number of previous abortions may be a potentially unmeasured confounder.

## Conclusion

This study suggested that a short IPI of 1 to 3 months did not appear to be significantly associated with adverse pregnancy outcomes. On the other hand, prolonging the IPI beyond 12 months might potentially correlate with a reduced likelihood of achieving a live birth. These findings offer evidence to patients and clinicians that there is no need to intentionally delay the first FET cycle after a normal menstrual cycle has resumed. However, extending the interval beyond 12 months could reduce the likelihood of successful subsequent pregnancies. Our findings should be interpreted with caution, given the single-center, retrospective nature of the study and the relatively small sample size of the study population. Further large-scale prospective studies, including studies in an the IVF setting, are needed to confirm our findings.

##  Supplemental Information

10.7717/peerj.20949/supp-1 Supplemental Information 1 Codebook

10.7717/peerj.20949/supp-2 Supplemental Information 2 Total data

10.7717/peerj.20949/supp-3 Supplemental Information 3 STROBE checklist

10.7717/peerj.20949/supp-4 Supplemental Information 4 Full results of the multivariable logistic regression model for live birth

10.7717/peerj.20949/supp-5 Supplemental Information 5 A sensitivity analysis using a minimally adjusted model for live birth

10.7717/peerj.20949/supp-6 Supplemental Information 6 Detailed Pregnancy Outcomes for Short IPI

10.7717/peerj.20949/supp-7 Supplemental Information 7 Results of Sensitivity Analysis for Short IPIs and Pregnancy Outcomes

## Additional Information and Declarations

### Data Availability

The following information was supplied regarding data availability: The raw data are available in the Supplemental Files.
