# Supplementary material for: Effect of the interpregnancy interval after early pregnancy loss on pregnancy outcomes after subsequent embryo transfer: a retrospective cohort study
Source: PeerJ. 2026 Mar 16;14:e20949. doi: 10.7717/peerj.20949 (PMC13001656; doi:10.7717/peerj.20949)
Supplement: Supplemental Information 4 [file peerj-14-20949-s004.docx]

| **Supplementary TableS 1. Full results of the multivariable logistic regression model for live birth** | | |
| --- | --- | --- |
| Variable | Adjusted Odds Ratio  (95% CI) | *P-value* |
| Interpregnancy Interval (IPI) |  |  |
| 3-6 months | 1.00 | - |
| <3 months | 1.001(0.61-1.63) | 0.996 |
| 6-12 months | 0.86(0.62-1.20) | 0.387 |
| ≥12 months | 0.55(0.32-0.93) | 0.027 |
| Female age at the time of OPU | 0.95（0.92-0.99） | 0.013 |
| BMI | 0.98（0.93-1.03） | 0.498 |
| No. of previous pregnancies and deliveries | 0.93（0.79-1.09） | 0.932 |
| No.of previous embryo transfer cycles | 0.92（0.74-1.14） | 0.463 |
| Diagnosis of PCOS | 0.83（0.44-1.54） | 0.558 |
| Gestational age at the preceding EPL | 0.99（0.98-1.006） | 0.434 |
| Means used to terminate the preceding EPL | 1.07（0.77-1.47） | 0.665 |
| Endometrial preparation protocols for FET | 1.26（0.89-1.80） | 0.185 |
| Endometrial thickness | 0.99（0.90-1.09） | 0.913 |
| Developmental stage of the transferred embryo | 0.97（0.60-1.58） | 0.927 |
| No.of embryos transferred | 1.59（1.02-2.46） | 0.038 |
| The transfer of ≥1 good-quality embryo | 1.77（1.28-2.433） | 0.0004 |
