# Supplementary material for: Effect of the interpregnancy interval after early pregnancy loss on pregnancy outcomes after subsequent embryo transfer: a retrospective cohort study
Source: PeerJ. 2026 Mar 16;14:e20949. doi: 10.7717/peerj.20949 (PMC13001656; doi:10.7717/peerj.20949)
Supplement: Supplemental Information 5 [file peerj-14-20949-s005.docx]

| **Supplementary Table S2. A sensitivity analysis using a minimally adjusted model for live birth** | | |
| --- | --- | --- |
| Variable | Fully adjusted OR  (95% CI, *P*) | Minimally adjusted OR (95%CI, *P*) |
| Interpregnancy Interval (IPI) |  |  |
| 3-6 months | 1.00 | 1.00 |
| 1~3 months | 1.001(0.61-1.63, 0.996) | 0.99(0.61- 1.60, 0.972) |
| 6-12 months | 0.86(0.62-1.20, 0.387) | 0.85(0.62- 1.17, 0.323) |
| ≥12 months | 0.55(0.32-0.93, 0.027) | 0.56(0.34- 0.93, 0.024) |

Fully adjusted model：adjusted for female age at the time of OPU, BMI, No. of previous pregnancies and deliveries, No. of previous embryo transfer cycles, diagnosis of PCOS, gestational age at the preceding EPL, means used to terminate the preceding EPL, endometrial preparation protocols for FET, endometrial thickness, developmental stage of the transferred embryo, No. of embryos transferred, and the transfer of ≥1 good-quality embryo).

Minimally adjusted model：adjusted only for maternal age and the transfer of ≥1 good-quality embryo.
