# Supplementary material for: Effect of the interpregnancy interval after early pregnancy loss on pregnancy outcomes after subsequent embryo transfer: a retrospective cohort study
Source: PeerJ. 2026 Mar 16;14:e20949. doi: 10.7717/peerj.20949 (PMC13001656; doi:10.7717/peerj.20949)
Supplement: Supplemental Information 6 [file peerj-14-20949-s006.docx]

| **Supplementary TableS3. Detailed Pregnancy Outcomes for Short IPI** | | |
| --- | --- | --- |
| **Outcome** | **IPI 1-2 months (n=8)** | **IPI 2-3 months (n=79)** |
| **Live birth, n/N (%)** | **4/8(50%)** | **32/79(40.5%)** |
| **Biochemical pregnancy, n/N (%)** | **4/8(50%)** | **45/79(56.9%)** |
| **Clinical pregnancy, n/N (%)** | **4/8(50%)** | **41/79(51.8%)** |
| **Clinical pregnancy loss, n/N (%)** | **0(0)** | **9/41(22.0%)** |
| **Preterm birth, n/N (%)** | **0(0)** | **4/32(12.5%)** |

*Note: Data are presented as descriptive statistics only. Formal statistical testing was not performed due to the small sample size in the 1-2 months IPI group.
