# Supplementary material for: Effect of the interpregnancy interval after early pregnancy loss on pregnancy outcomes after subsequent embryo transfer: a retrospective cohort study
Source: PeerJ. 2026 Mar 16;14:e20949. doi: 10.7717/peerj.20949 (PMC13001656; doi:10.7717/peerj.20949)
Supplement: Supplemental Information 7 [file peerj-14-20949-s007.docx]

| **Supplementary Table S4. Results of Sensitivity Analysis for Short IPIs and Pregnancy Outcomes** | | | | |
| --- | --- | --- | --- | --- |
| **Outcome** | **IPI Group** | **n/N (%)** | **aOR (95% CI)** | **P-value** |
| **Live birth** | **3-6 months** | 167/402(41.5%) | 1 [Reference] |  |
|  | **2-3 months** | **32/79(40.5%)** | **0.94(0.56-1.57)** | **0.813** |
| **Biochemical pregnancy** | **3-6 months** | 232/402(57.7%) | 1 [Reference] |  |
|  | **2-3 months** | **45/79(56.9%)** | **0.95(0.57-1.578)** | **0.835** |
| **Clinical pregnancy** | **3-6 months** | 208/402(51.7%) | 1 [Reference] |  |
|  | **2-3 months** | **41/79(51.8%)** | **0.94(0.57-1.56)** | **0.816** |
| **Clinical pregnancy loss** | **3-6 months** | 41/208(19.7%) | 1 [Reference] |  |
|  | **2-3 months** | **9/41(22.0%)** | **1.003(0.46-2.20)** | **0.992** |
| **Preterm birth** | **3-6 months** | 22/167(13.2%) | 1 [Reference] |  |
|  | **2-3 months** | **4/32(12.5%)** | **0.98(0.32-2.99)** | **0.968** |

adjusted for female age at the time of OPU, BMI, No. of previous pregnancies and deliveries, No.of previous embryo transfer cycles, diagnosis of PCOS, gestational age at the preceding EPL, means used to terminate the preceding EPL, endometrial preparation protocols for FET, endometrial thickness, developmental stage of the transferred embryo, No.of embryos transferred, and the transfer of ≥1 good-quality embryo.
